# Comparison between whole-body MRI and Fluorine-18-Fluorodeoxyglucose PET or PET/CT in oncology: a systematic review

**DOI:** 10.2478/raon-2013-0007

**Published:** 2013-07-30

**Authors:** Mario Ciliberto, Fabio Maggi, Giorgio Treglia, Federico Padovano, Lucio Calandriello, Alessandro Giordano, Lorenzo Bonomo

**Affiliations:** 1 Institute of Radiology, Università Cattolica del Sacro Cuore, Rome, Italy; 2 Institute of Nuclear Medicine, Università Cattolica del Sacro Cuore, Rome, Italy

**Keywords:** positron emission tomography, PET/CT, fluorodeoxyglucose, whole-body magnetic resonance imaging, diffusion-weighted imaging, oncology

## Abstract

**Background:**

The aim of the article is to systematically review published data about the comparison between positron emission tomography (PET) or PET/computed tomography (PET/CT) using Fluorine-18-Fluorodeoxyglucose (FDG) and whole-body magnetic resonance imaging (WB-MRI) in patients with different tumours.

**Methods:**

A comprehensive literature search of studies published in PubMed/MEDLINE, Scopus and Embase databases through April 2012 and regarding the comparison between FDG-PET or PET/CT and WB-MRI in patients with various tumours was carried out.

**Results:**

Forty-four articles comprising 2287 patients were retrieved in full-text version, included and discussed in this systematic review. Several articles evaluated mixed tumours with both diagnostic methods. Concerning the specific tumour types, more evidence exists for lymphomas, bone tumours, head and neck tumours and lung tumours, whereas there is less evidence for other tumour types.

**Conclusions:**

Overall, based on the literature findings, WB-MRI seems to be a valid alternative method compared to PET/CT in oncology. Further larger prospective studies and in particular cost-effectiveness analysis comparing these two whole-body imaging techniques are needed to better assess the role of WB-MRI compared to FDG-PET or PET/CT in specific tumour types.

## Introduction

Accurate staging and thorough tumour surveillance are essential in patients with a neoplastic disease to assess prognosis and to decide the most appropriate therapeutic options. Imaging plays a key role in these evaluation steps: multi-slice computed tomography (CT) and, recently, positron emission tomography/CT (PET/CT) are widely used in order to get an integrated diagnostic approach to cancer as a systemic disease.^1^ In particular, the use of Fluorine-18-Fluorodeoxyglucose (FDG) tracer made, up to now, PET contribution to oncologic imaging matchless by any other functional imaging modality.^2^ However, this technique uses ionizing radiations and has some limitations for what concerns spatial and contrast resolution; false positive and false negative results of FDG-PET are well known, too.

Magnetic resonance imaging (MRI), with its lack of ionizing radiation, high soft tissue contrast and good spatial resolution, is a useful application for tumour detection and staging of malignancies and could overcome the limits of FDG-PET/CT.^3^

In recent years, significant improvements in hardware and important innovations in sequence design and image acquisition have allowed a whole-body imaging with MRI in a suitable acquisition time without impairment of spatial resolution.^4^ Furthermore, the introduction of diffusion-weighted MRI (DWI) has increased the potential for the detection of malignancies throughout the body.^5^ Whole body MRI (WB-MRI) has then emerged as an excellent candidate for staging and surveillance of patients with neoplastic disease and many authors have compared FDG-PET/CT and WB-MRI in oncology.

Our article aims to systematically review the current evidence on the comparison between PET or PET/CT using FDG and WB-MRI in patients with different tumours.

## Methods

A comprehensive literature search of studies published in PubMed/MEDLINE, Scopus and Embase databases was carried out to find relevant peer-reviewed articles on the comparison of FDG-PET or PET/CT and WB-MRI in patients with different tumours.

A search algorithm based on a combination of the terms: a) “PET” OR “positron emission tomography” AND b) “whole body MR” OR “whole-body MR” OR “whole body magnetic resonance” OR “whole-body magnetic resonance” OR “whole body MRI” OR “whole-body MRI” was used. No beginning date limit was used and the search was updated until April 2012.

All the studies which compared FDG-PET or PET/CT and WB-MRI in oncology were considered eligible for inclusion in this systematic review.

The exclusion criteria were: a) articles not within the field of interest of this review; b) review articles, editorials or letters, comments, conference proceedings; c) case reports or small case series (less than seven patients included); d) articles not in English, Spanish, French or German language; e) possible data overlap (in this case the most complete article was included).

Two researchers (MC and GT) reviewed the titles and the abstracts of the retrieved articles, applying the inclusion and exclusion criteria mentioned above. The full-text version of the retrieved articles was reviewed to confirm their eligibility for inclusion. Disagreements were resolved in a consensus meeting.

For each included study, information was collected concerning basic study (authors, journal, year of publication, country of origin, type of study), patient characteristics (number of patients, mean age, gender and type of tumours evaluated), methodological aspects about PET imaging (device used, injected activity, time between tracer injection and image acquisition, PET acquisition protocol, image analysis), methodological aspects about WB-MRI (field strength, sequences used, slice thickness, contrast media, diffusion-weighted imaging, apparent diffusion coefficient, acquisition time) and reference standard used.

## Results

### Literature search

The comprehensive literature search revealed 688 articles. Reviewing titles and abstracts, 644 articles were excluded applying the criteria mentioned above: 564 studies were excluded because not within the range of interest of this review; 69 articles were excluded because review articles, editorials or letters, comments, conference proceedings; 4 articles were excluded because case reports or small case series (less than seven patients included)^6^–^9^; 2 articles were excluded because not in English, Spanish, French or German language^10^,^11^; 5 articles were excluded for possible data overlap.^12^–^16^

**FIGURE 1.**
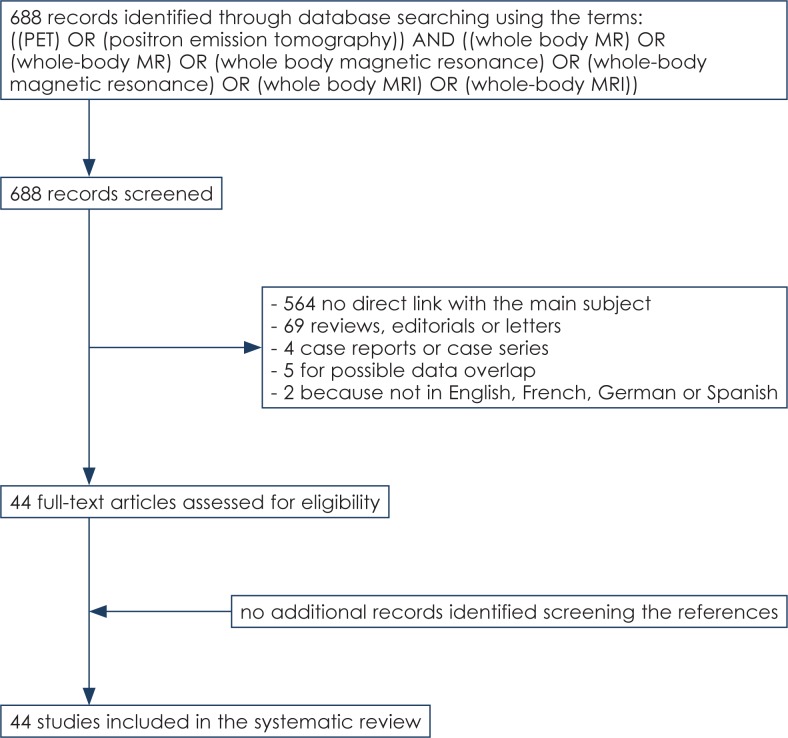
Flow chart of the search for eligible studies on the comparison of FDG-PET or PET/CT and WB-MRI in oncology.

Lastly, forty-four articles comprising 2287 patients were retrieved in full-text version and included in this systematic review (Figure 1).^17^–^60^ No additional studies were found screening the references of these articles.^6^–^16^ The characteristics of the included studies are presented in Tables 1–3.

Mixed tumours were evaluated in 12 articles^17^–^28^, lymphomas in 7^29^–^35^; bone tumours in 7^36^–^42^; head and neck tumours in 5^43^–^47^; lung tumours in 4^48^–^51^; melanoma in 3^52^–^54^; breast cancer in 2^55^,^56^; colorectal tumours in 2^57^,^58^; neuroendocrine tumours in 2.^59^,^60^

### Literature data discussion

#### Mixed tumours

First of all, Antoch *et al*.^17^ performing both FDG-PET/CT and WB-MRI in 98 patients with different malignancies, recommended the use of FDG-PET/CT as first-line whole-body imaging modality for tumour staging. In fact, the overall TNM stage was correctly determined in 75 cases with PET/CT (77%) and in 53 with WB-MRI (54%). Compared with WB-MRI, PET/CT had a direct impact on patient management in 12 patients. WB-MRI findings changed the therapy regimen in 2 patients compared with PET/CT.^17^

In 2005, Schmidt *et al*.^18^ evaluating 41 patients with mixed tumours using both methods found that WB-MRI was highly sensitive in detecting distant metastases (sensitivity was 96% for WB-MRI and 82% for FDG-PET/CT; specificity was 82% for both methods), whereas PET/CT was superior in lymph node staging (sensitivity was 98% for PET/CT and 80% for WB-MRI; specificity was 83% for PET/CT and 75% for WB-MRI). Accuracy for correct TNM staging was 96% for PET/CT and 91% for WB-MRI.^18^

In 2007 Komori *et al*.^19^ comparing FDG-PET/CT and DWI WB-MRI in 16 patients with malignant tumours reported that DWI WB-MRI may be useful in detecting malignancies, even if differentiating malignant and benign tumours may be difficult with this method. Twenty-five (92.6%) of the 27 malignant lesions were detected by DWI WBMRI whereas 22 malignant tumours (81.5%) were detected by FDG-PET/CT.^19^

Also Li *et al*.^20^ reported that DWI WB-MRI is a feasible imaging method in oncology, providing comparable results to PET imaging in 30 oncologic patients evaluated.

Brauck *et al*.^21^ evaluated a WB-MRI protocol by using unenhanced T2-weighted and contrast-enhanced T1-weighted real-time sequences during continuous table movement in 11 patients with FDG-PET/CT positive for metastases. Seventy-three of 75 metastases detected by PET/CT were correctly diagnosed by using WB-MRI, demonstrating the feasibility of this method in detecting metastases.^21^

In 2008, Yang *et al*.^22^ evaluated 56 patients with different tumours demonstrating the valuable role of DWI WB-MRI in tumour detection. Twelve patients underwent also FDG-PET. Among the diagnostic imaging methods DWI WB-MRI showed the highest sensitivity and specificity in detecting bone metastases. Among the twelve results compared with PET, eight were identical (concordance of 66.7%), one was found to be false-positive at MRI, two were found false-negative at MRI, one case was false-negative at PET and true-positive at MRI.^22^

In 2009, Stecco *et al*.^23^ compared FDG-PET/CT and DWI WB-MRI in staging 29 oncologic patients. Using FDG-PET/CT as reference standard, DWI WB-MRI interpreted by two readers had a sensitivity of 87–89%, a specificity of 98–99%, and an accuracy of 98–99%. These authors underlined the usefulness of DWI WB-MRI in cancer screening, staging, restaging and follow-up.^23^

Krohmer *et al*.^24^ evaluated 24 paediatric tumours with WB-MRI and FDG-PET, showing that WBMRI had high sensitivity for the detection of malignant disease. Overall 190 lesions were detected by WB-MRI and 155 lesions were found by FDG-PET. In patients with suspected bone lesions, WB-MRI should be considered for initial disease evaluation prior to specific and regional imaging methods to reduce the overall number of imaging examinations and radiation exposure.^24^

In 2011, Fischer *et al*.^25^ prospectively evaluated the diagnostic accuracy of WB-MRI with and without DWI compared with PET/CT (as reference standard) in 66 oncologic patients. PET/CT revealed 374 malignant lesions in 48/64 (75%) patients. Detection rates of WB-MRI with and without DWI were 84% and 64%, respectively. The detection rate was significantly higher with side-by-side analysis and fused image analysis compared with WB-MRI alone.^25^

Recently, Schmidt *et al*.^26^ demonstrated that both FDG-PET/CT and WB-MRI were efficient diagnostic triage methods in 135 patients planned for radioembolisation of liver metastases. Overall, FDG-PET/CT showed a higher diagnostic accuracy compared to WB-MRI. Both modalities, combined, exhibited high sensitivity for the diagnosis of extra-hepatic tumour manifestations. Patient-based sensitivity for detection of extra-hepatic disease was 94% for PET/CT and 91% for WB-MRI. Overall, by combining both modalities, the specificity for inclusion to radioembolisation therapy was 99%.^26^

Cafagna *et al*.^27^ evaluating 38 cancer patients demonstrated that DWI WB-MRI may be used in detecting tumours but is less effective in characterizing lymph nodal and bone lesions compared to FDG-PET/CT. The qualitative analysis of DWI WB-MRI and FDG-PET/CT showed that two patients were negative at both techniques. DWI WB-MRI was positive in 36 patients, 34 of whom were positive and two negative at FDG-PET/CT, respectively. A significant discordance was found between the two methods (255 lesions were identified by DWI WB-MRI and 184 by FDG-PET/CT).^27^

Lastly, Manenti *et al*.^28^ reported that DWI WB-MRI should be considered as alternative tool to conventional whole-body methods for tumour staging in cancer patients. Evaluating 45 patients using both methods, detection rates of malignancy did not differ between DWI WB-MRI and FDG-PET/CT.^28^

#### Lymphomas

##### Staging

Punwani *et al*.^29^ evaluated 31 subjects with lymphoma using both WB-MRI and enhanced FDG-PET/CT (used as reference standard) demonstrating that WB-MRI can accurately depict nodal and extranodal disease and may provide an alternative non-ionizing imaging method for initial staging. WB-MRI and enhanced PET/CT showed a good agreement for nodal and extranodal staging. The sensitivity and specificity of WB-MRI were 98% and 99%, respectively, for nodal disease; 91% and 99%, respectively, for extranodal disease.^29^

van Ufford *et al*.^30^ compared DWI WB-MRI with FDG-PET/CT in the staging of 22 patients with newly diagnosed lymphoma. These authors found a moderate overall agreement between DWI WB-MRI and FDG-PET/CT. Ann Arbor staging, according to DWI WB-MRI findings, was concordant with that of FDG PET/CT findings in 77% (17/22) of patients. In the care of patients with newly diagnosed lymphoma, staging with DWI WB-MRI did not result in underestimation of stage relative to the results with FDG-PET/CT. In a minority of patients, reliance on DWI WB-MRI led to clinically important overstaging relative to the results with FDG-PET/CT.^30^

Recently, Abdulqadhr *et al*.^31^ compared DWI WB-MRI with FDG-PET/CT in the staging of 31 lymphoma patients (8 with Hodgkin’s lymphoma and 23 with non-Hodgkin’s lymphomas). The staging was the same for DWI WB-MRI and FDG-PET/CT in 28 (90.3%) patients and different in three (9.7%). No Hodgkin lymphoma or aggressive non-Hodgkin’s lymphoma patients had different staging using both methods. Three indolent lymphocytic lymphomas had higher staging with DWI WB-MRI when compared with FDG-PET/CT.^31^

Gu *et al*.^32^ evaluated the diagnostic performance of WB-MRI with or without DWI in the detection of 17 patients with newly diagnosed lymphomas, using FDG-PET/CT as the reference standard. The addition of DWI to conventional WB-MRI improved diagnostic accuracy for lymphomas. These authors suggested that WB-MRI could be useful as an alternative method to FDG-PET/CT in the management of lymphomas.^32^

##### Treatment response assessment

Lin *et al*.^33^ assessed post-treatment changes in 15 patients with diffuse large B-cell lymphomas on DWI WB-MRI using PET/CT as the reference standard. After chemotherapy, among 85 examined lymph nodal regions, residual nodes were present in 62 (73%) regions on DWI WB-MRI. Of these 62 regions, 26 had persistent lymph nodes with longest transverse diameter > 10mm (MRI size criteria for positivity). Only 6 of these 26 regions were considered positive on PET/CT. DWI with ADC mapping showed a significant increase in ADC values of residual masses persisting after treatment and were helpful to assess the treatment response in patients with diffuse large B-cell lymphomas.^33^

Wu *et al*.^34^ evaluated the feasibility of DWI WB-MRI in the early chemotherapeutic response assessment of 8 patients with large B-cell lymphomas. These authors found that the results of WB-MRI with or without DWI were comparable with those of FDG-PET/CT.^34^

Recently, Chen *et al*.^35^ reported that DWI WB-MRI, combined with the dynamic changes of ADC value, was a valid alternative method compared to FDG PET/CT in assessing treatment response to chemotherapy in 10 patients with non-Hodgkin’s lymphoma.^35^

#### Bone tumours

##### Primary tumours

Shortt *et al*.^36^ found that WB-MRI performed better than FDG-PET/CT in the assessment of disease activity in 24 patients with multiple myeloma. FDG-PET/CT had a sensitivity of 59%, specificity of 75%, and accuracy of 65%. WB-MRI had a sensitivity of 68%, specificity of 83% and accuracy of 74%. In 62% of cases, FDG-PET/CT and WB-MRI findings were concordant. When PET and WB-MRI findings were concordant and positive, specificity was 100%.^36^

##### Bone metastases

Daldrup-Link *et al*.^37^ compared the diagnostic accuracy of WB-MRI and FDG-PET for the detection of bone metastases in 39 children. Sensitivity for the detection of bone metastases were 90% for FDG-PET and 82% for WB-MRI.^37^

In 2007, Schmidt *et al*.^38^ prospectively compared the diagnostic accuracy of WB-MRI and FDG-PET/CT for the detection of bone metastases in 30 patients with different oncologic diseases. WB-MRI showed a sensitivity, specificity and accuracy of 94%, 76% and 91%, respectively. PET/CT achieved a sensitivity, specificity and accuracy of 78%, 80% and 78%, respectively. Cut-off size for the detection of malignant bone lesions was 2 mm for WB-MRI and 5 mm for PET/CT.^38^

In 2008, Ribrag *et al*.^39^ suggested that non-invasive morphological procedures (WB-MRI and FDG-PET/CT) could be superior to bone marrow biopsy for bone marrow assessment in aggressive lymphomas. Both WB-MRI and PET/CT detected bone marrow lesions in the 9/43 patients, but two patients with multiple lesions had more lesions detected by PET/CT compared to MRI.^39^

Kumar *et al*.^40^ compared WB-MRI and FDG-PET/CT for the detection of bone marrow metastases in 26 children with small-cell neoplasms. WB-MRI showed a sensitivity, specificity and accuracy of 97.5%, 99.4%, and 99% respectively. FDG-PET/CT showed a sensitivity, specificity and accuracy of 90.0%, 100%, and 98%. Both WB-MRI and FDG-PET/CT showed excellent agreement with the final diagnosis.^40^

In 2009, Takenaka *et al*.^41^ prospectively compared WB-MRI (with and without DWI) and FDG-PET/CT in the detection of bone metastases in 115 patients with non-small cell lung cancer. These authors suggested that DWI WB-MRI can be used for bone metastases assessment in patients with non-small cell lung cancer being more accurate than bone scintigraphy and FDG-PET/CT.^41^

Recently, Heusner *et al*.^42^ found that FDG-PET/CT and WB-MRI were equally suitable for the detection of bone metastases in 109 patients with non-small cell lung cancer and malignant melanoma. The sensitivity, specificity, and accuracy for the detection of bone metastases was 45%, 99%, and 94% with FDG-PET/CT and 64%, 94%, and 91% with WB-MRI.^42^

#### Head and neck tumours

In 2010, Ng *et al*.^43^ prospectively compared WB-MRI and FDG-PET/CT for the detection of residual/recurrent nasopharyngeal carcinoma in 179 patients. On a per patient-based analysis, sensitivity and specificity of WB-MRI were similar to those of FDG-PET/CT (90.9% *vs*. 87.3%, and 91.1% vs. 90.3%, respectively). A combined interpretation of both methods increased the sensitivity to 94.5%.^43^

In the same year, O’Neill *et al*.^44^ compared WB-MRI and FDG-PET/CT for the staging of 15 patients with head and neck tumours. This study found radiological staging discordance between the two imaging modalities: T-staging showed a 74% of concordance, N-staging a 80% of concordance and M-stage a 100% of concordance.^44^

Recently, Ng *et al*.^45^ compared WB-MRI and FDG-PET/CT in 79 treated oropharyngeal or hypopharyngeal squamous cell carcinoma. PET/CT showed a trend towards higher diagnostic accuracy than WB-MRI in detecting residual/recurrent tumours or second primary tumours. The combined use of PET/CT and WB-MRI provided more added value to WB-MRI alone than to PET/CT alone. Sensitivity and specificity of FDG-PET/CT on a patient-based analysis were 72% and 94%. Sensitivity and specificity of WB-MRI on a patient-based analysis were 55% and 90%.^45^

The same group prospectively compared the diagnostic value of FDG-PET/CT and WB-MRI for the assessment of distant metastases and second primary cancers in 103 patients with untreated oropharyngeal or hypopharyngeal squamous cell carcinoma. Again, FDG-PET/CT showed a consistent trend toward higher sensitivity compared to WB-MRI for the detection of distant metastases and secondary primary cancers in these patients.^46^

Lastly, Eiber *et al*.^47^ reported that a combination of FDG-PET/CT and WB-MRI increased the diagnostic accuracy in the staging of 20 patients with head and neck tumours.^47^

#### Lung cancer

In 2008, Plathow *et al*.^48^ evaluated and compared FDG-PET/CT with WB-MRI in the correct staging of 52 patients with advanced non-small cell lung cancer (NSCLC). In the correct staging of advanced NSCLC, PET/CT had advantages in N-staging, whereas WB-MRI had certain advantages in T-staging. WB-MRI correctly T-staged all patients. PET/CT did not correctly stage chest wall infiltration in 4 cases (sensitivity: 92.3%; specificity: 100%). PET/CT correctly N-staged 51 patients (sensitivity: 96.1%; specificity: 100%). WB-MRI showed a significant tendency to understage N-status (sensitivity: 88.5%; specificity: 96.1%). In 2 patients, distant metastases were detected by both techniques.^48^

In the same year, Ohno *et al*.^49^ prospectively compared WB-MRI with and without DWI and FDG-PET/CT for M-stage assessment in 203 NSCLC patients. These authors found that DWI WB-MRI can be used for M-stage assessment in NSCLC patients with accuracy as good as that of PET/CT. The area under the ROC curve was 0.89 for FDG-PET/CT, 0.85 for DWI WB-MRI and 0.81 for WB-MRI without DWI, excluding brain metastases (due to the low accuracy of FDG-PET/CT in detecting brain metastases).^49^

Yi *et al*.^51^ prospectively compared the diagnostic accuracy of FDG-PET/CT and WB-MRI for TNM stage of 165 patients with NSCLC. WB-MRI was more useful for detecting brain and hepatic metastases, whereas PET/CT was more useful for detecting lymph node and soft-tissue metastases. Primary tumours (n=123 patients) were correctly staged in 101 (82%) patients at PET/CT and in 106 (86%) patients at WB-MRI. N stages (n=150 patients) were correctly determined in 105 (70%) patients at PET/CT and in 102 (68%) patients at WB-MRI. Thirty-one (20%) of 154 patients had metastatic lesions. Accuracy for detecting metastases was comparable between PET/CT and WB-MRI (86%). WB-MRI was more useful for detecting brain and hepatic metastases, whereas PET/CT was more useful for detecting lymph node and soft-tissue metastases.^50^

Chen *et al*.^51^ compared the diagnostic accuracy of DWI WB-MRI and FDG-PET/CT for assessment of 56 NSCLC patients. DWI WB-MRI was a feasible imaging method for the assessment of lymph nodal and metastatic spread with high accuracy, but it was limited in the evaluation of neck lymph nodal metastases and small metastatic lung nodules. Primary tumours were correctly detected in 56 (100%) patients by both PET/CT and DWI WB-MRI. Sensitivity, specificity and accuracy for lymph nodal metastases were 91%, 90% and 90% with DWI WB-MRI and 98%, 97% and 97% with PET/CT, respectively. Sensitivity, specificity and accuracy for other metastases were 90%, 95% and 92% with DWI WB-MRI and 98%, 100% and 98% with PET/CT.^51^

#### Melanoma

Pfannenberg *et al*.^52^ compared the diagnostic accuracy and impact on patient management of FDG-PET/CT and WB-MRI in staging of 64 patients with advanced melanoma. The overall accuracy of PET/CT was 86.7% compared to 78.8% for WB-MRI. PET/CT was significantly more accurate in N-staging and in detecting skin and subcutaneous metastases, whereas WB-MRI was more sensitive in detecting liver, bone and brain metastases. WB-MRI was less sensitive but more specific than PET/CT in classifying pulmonary lesions.^52^

Laurent *et al*.^53^ compared WB-MRI (with and without DWI) and FDG-PET/CT for staging of 35 patients with advanced melanoma. The sensitivity and specificity for WB-MRI without DWI were 82% and 97%, respectively, while for PET/CT were 72.8% and 92.7%, respectively. DWI allowed the detection of 14 supplementary malignant lesions (20%) in comparison with standard MRI protocol.^51^ In particular WB-MRI has been shown to be the most accurate method for detecting metastases in the liver, bone, subcutaneous and intra-peritoneal sites.^53^

Recently, Dellestable *et al*.^54^ found that DWI WB-MRI was superior compared to FDG-PET/CT in the staging of 40 patients with melanoma. Sensitivity and specificity were 74% and 89% for FDG-PET/CT, 83% and 96% for DWI WB-MRI. The sensitivity of MRI was distinctly superior compared to that of PET/CT for both hepatic and pulmonary lesions.^54^

#### Breast cancer

Schmidt *et al*.^55^ compared the diagnostic accuracy of WB-MRI and FDG-PET/CT for the detection of tumour recurrence in 33 patients with breast cancer. WB-MRI and PET/CT were both useful for the detection of tumour recurrence. WB-MRI was highly sensitive to detect distant metastatic disease. PET/CT was more sensitive in detecting lymph node involvement. Overall sensitivity was 91% for PET/CT and 90% for WB-MRI. Overall specificity was 90% for FDG-PET/CT and 86% for WB-MRI.^55^

Heusner *et al*.^56^ prospectively compared the diagnostic value of DWI WB-MRI and FDG-PET/CT for breast cancer staging in 20 patients. DWI resulted a sensitive but unspecific method for the detection of locoregional or metastatic breast cancer. These authors suggested that DWI WB-MRI is not alternative to FDG-PET/CT in staging breast cancer. The sensitivity, specificity, and accuracy for FDG-PET/CT were 94%, 99%, and 98%, respectively, whereas for DWI WB-MRI were 91%, 72%, and 76%, respectively.^56^

#### Colorectal cancer

Squillaci *et al*.^57^ assessed the accuracy of WB-MRI in comparison with FDG-PET/CT in staging 20 patients with colorectal carcinoma. These authors found that WB-MRI was a feasible method for staging colorectal cancer but could not substitute PET/CT. Lymph-nodal metastases were detected in 10/20 cases at WB-MRI and in 15/20 at PET/CT. M-stage was evaluated for liver metastases (27 lesions detected in 15 patients with WB/MRI; 23 lesions detected in 15 patients with PET/CT), lung metastases (19 lesions detected in 5 patients with WB-MRI, 25 lesions detected in 7 patients with PET/CT), and bone (9 lesions detected in 3 patients with both methods).^57^

Schmidt *et al*.^58^ assessed the diagnostic accuracy of WB-MRI compared with FDG-PET/CT in the follow-up of 24 patients suffering from colorectal cancer. Malignant foci were detected in 71% of patients with both methods. Lymph nodal metastases were better detected using PET/CT (sensitivity was 93% for PET/CT and 63% for WB-MRI), whereas distant metastases were depicted equally well by both investigations (sensitivity was 80% for PET/CT and 78% for WB-MRI). Overall sensitivity, specificity and diagnostic accuracy was 86%, 96% and 91% for PET/CT, and 72%, 93% and 83% for WB-MRI.^58^

#### Neuroendocrine tumours

Giraudet *et al*.^59^ comparing FDG-PET/CT and WB-MRI in 50 patients with suspected recurrent medullary thyroid carcinoma found a superior diagnostic accuracy of WB-MRI compared to FDG-PET/CT.^59^

Takano *et al*.^60^ found that DWI WB-MRI had a higher detection rate of metastatic lesions in 11 patients with paraganglioma when compared with metaiodobenzylguanidine scintigraphy or FDG-PET, particularly for lymph nodal and liver metastases. The limitations of DWI WB-MRI were possible false-positive findings and lower detectability of mediastinal lymph nodes and lung metastases.^60^

#### General remarks and conclusions

On the basis of our systematic review, we found several articles in which mixed tumour types were evaluated using both imaging methods.^17^–^28^ For what concerns the specific tumour types, more evidence exists for lymphomas^29^–^35^, bone tumours^36^–^42^, head and neck tumours^43^–^47^ and lung tumours^48^–^51^, whereas there is less evidence for other tumour types.

Overall, based on the literature findings, WB-MRI seems to be a valid alternative method compared to PET/CT in oncology. Nevertheless, it should be considered that the studies included in this systematic review were highly heterogeneous not only about the patient population evaluated (Table 1), but also for those technical aspects related to PET imaging and WB-MRI (Table 2). In particular, DWI, when performed, seemed to provide an added value to WB-MRI compared to FDG-PET/CT, increasing the sensitivity (due to a better lesion to background contrast).

A possible limitation of some studies evaluated in this systematic review is the reference standard used. In fact, in some articles the diagnostic performance of WB-MRI was assessed considering PET or PET/CT as a reference standard. This is a possible source of bias, because FDG-PET or PET/CT has its own limitations, mainly due to the possibility of false-positive or false-negative results, which could affect the diagnostic accuracy calculated for WB-MRI (Table 3).

Possible advantages of WB-MRI compared to FDG-PET or PET/CT are: the lack of ionizing radiation, the higher soft-tissue contrast, the higher spatial resolution, the better assessment of non FDG-avid tumour types or sites of physiological FDG uptake. On the other hand, it should be considered that WB-MRI has a longer examination time compared to PET/CT and more variable acquisition protocols.

Both these imaging techniques still show limited worldwide availability if compared to other conventional imaging methods.

Referring to the costs, Plathow *et al*.^61^, performing a cost-analysis study, demonstrated that both whole-body imaging techniques allow substantial reduction of health care costs in many tumour types. On the basis of a simple full cost analysis, total costs of whole-body PET/CT were higher than those of whole-body MRI by a factor of about 2.0.^61^

Further larger prospective studies and in particular cost-effectiveness analysis comparing these two whole-body imaging techniques is needed to better assess the role of WB-MRI compared to FDG-PET or PET/CT in specific tumour types. Furthermore, emerging hybrid PET/MRI devices will increase the number of studies comparing PET to WB-MRI.

## Figures and Tables

**TABLE 1.** Basic studies and patient characteristics

**Authors**	**Year**	**Country**	**Study type**	**No. of patients**	**Mean Age**	**% Male**	**Type of tumors**
Antoch *et al*.^17^	2003	Germany	Prospective	98	58	64%	Mixed
Schmidt *et al*.^18^	2005	Germany	Prospective	41	56	44%	Mixed
Komori *et al.*^19^	2007	Japan	NR	16	66	70%	Mixed
Li *et al*.^20^[18]	2007	China	NR	30	48	37%	Mixed
Brauck *et al*.^21^	2008	Germany	Prospective	11	53	63%	Mixed
Yang *et al*.^22^	2008	China	NR	56	57	71%	Mixed
Stecco *et al*.^23^	2009	Italy	Prospective	29	NR	NR	Mixed
Krohmer *et al*.^24^	2010	Germany	Prospective	24	11	NR	Mixed
Fischer *et al*.^25^	2011	Switzerland	Prospective	66	60	66%	Mixed
Schmidt *et al*.^26^	2012	Germany	Retrospective	135	61	45%	Mixed
Cafagna *et al*.^27^	2012	Italy	Retrospective	38	60	47%	Mixed
Manenti *et al*.^28^	2012	Italy	Retrospective	45	66	53%	Mixed
Punwani *et al*.^29^	2010	England	NR	31	13	58%	Lymphoma
van Ufford *et al*.^30^	2011	Netherlands	Prospective	22	49	68%	Lymphoma
Abdulqadhr *et al*.^31^	2011	Sweden	Prospective	31	47	64%	Lymphoma
Gu *et al*.^32^	2011	China	NR	17	50	65%	Lymphoma
Lin *et al*.^33^	2011	France	Prospective	15	48	60%	Lymphoma
Wu *et al*.^34^	2011	Finland	Prospective	8	54	50%	Lymphoma
Chen *et al*.^35^	2012	China	Prospective	10	45	40%	Lymphoma
Shortt *et al*.^36^	2009	Ireland	NR	24	67	46%	Multiple Mieloma
Daldrup-Link *et al*.^37^	2001	Germany	NR	39	13	69%	Bone
Schmidt *et al*.^38^	2007	Germany	Prospective	30	58	60%	Bone
Ribrag *et al*.^39^	2008	France	Prospective	47	50	50%	Bone
Kumar *et al*.^40^	2008	India	NR	26	NR	NR	Bone
Takenaka *et al*.^41^	2009	Japan	Prospective	115	72	57%	Bone
Heusner *et al*.^42^	2011	Germany	Prospective	109	57	60%	Bone
Ng *et al*.^43^	2010	Taiwan	Prospective	179	47	75%	Head and neck
O‘Neill *et al*.^44^	2010	Ireland	Prospective	15	59	66%	Head and neck
Ng *et al*.^45^	2011	Taiwan	Prospective	79	52	88%	Head and neck
Chan *et al*.^46^	2011	Taiwan	Prospective	103	53	94%	Head and neck
Eiber *et al*.^47^	2012	Germany	Prospective	20	56	80%	Head and neck
Plathow *et al*.^48^	2008	Germany	NR	52	62	69%	Lung
Ohno *et al*.^49^	2008	Japan	Prospective	203	72	53%	Lung
Yi *et al*.^50^	2008	Korea	Prospective	165	61	72%	Lung
Chen *et al*.^51^	2010	China	NR	56	51	62%	Lung
Pfannenberg *et al*.^52^	2007	Germany	Prospective	64	58	41%	Melanoma
Laurent *et al*.^53^	2010	France	Prospective	35	NR	NR	Melanoma
Dellestable *et al*.^54^	2011	France	Prospective	40	57	50%	Melanoma
Schmidt *et al*.^55^	2008	Germany	NR	33	55	0%	Breast
Heusner *et al*.^56^	2010	Germany	Prospective	20	54	0%	Breast
Squillaci *et al*.^57^	2008	Italy	NR	20	56	60%	Colorectal
Schmidt *et al*.^58^	2009	Germany	Retrospective	24	62	NR	Colorectal
Giraudet *et al*.^59^	2007	France	Prospective	55	56	62%	Neuroendocrine tumors
Takano *et al*.^60^	2008	Japan	Prospective	11	40	55%	Neuroendocrine tumors

NR = not reported

**TABLE 2.** Technical aspects of the included studies

**Authors**	**Device activity**	**Injected**	**Time between tracer injection and image acquisition (min)**	**PET acquisition protocol**	**Image analysis**	**Field strenght (T)**	**Sequences used**	**Slice thickness**	**Contrast media administration**	**DWI**	**ADC**	**Acquisition time (min)**	**Reference standard**
Antoch *et al*.^17^	PET/CT	350 MBq	60	Static acquisition (3–5min per bed position)	Qualitative	1.5	T1w(chest, abdomen), T2w(chest, abdomen), T1w(chest, abdomen)after CM, T2w(chest, abdomen)after CM	7mm	Yes	No	No	26	Histology and/or follow up
Schmidt *et al*.^18^	PET/CT	200 MBq	60	Static acquisition (3min per bed position)	Qualitative, semi-quantitative	1.5	STIR(WB), HASTE(chest), T1w(WB), 3D-VIBE(abdomen, pelvis)after CM	5mm	Yes	No	No	55	Histology and PET/CT
Komori *et al*.^19^	PET/CT	3.7 MBq/kg	60	Static acquisition	Qualitative, semi-quantitative	1.5	DW-EPI(WB)	6mm	No	Yes (Bvalue0-1000mm2/s)	Yes	9	Histology and/or follow up
Li *et al*.^20^	PET	NR	NR	Static aquisition	Qualitative, semi-quantitative	1.5	DW-EPI-STIR(WB)	7mm	No	Yes (Bvalue0-800mm2/s)	Yes	30	Follow up
Brauck *et al*.^21^	PET/CT	300–340 MBq	60	Static aquisition	Qualitative	1.5	T1wSSFP(WB), T1wSSFP(WB) after CM, T2wSSFP(WB)	5mm	Yes	No	No	6	PET/CT
Yang *et al*.^22^	PET	NR	NR	Static acquisition	Qualitative	1.5	DW-EPI-STIR(WB)	7mm	No	Yes (Bvalue0-400-600mm2/s)	No	17–21	Follow up
Stecco *et al*.^23^	PET/CT	3.5 MBq/kg	60	Static acquisition	Qualitative, semi-quantitative	1.5	DW-EPI-STIR(WB)	5mm	No	Yes (Bvalue0-500-1000mm2/s)	No	20	PET/CT
Krohmer *et al*.^24^	PET	NR	NR	Static aquisition	Qualitative	1.5	T2w-STIR(WB), T1wTSE(WB)	6–8mm	No	No	No	45	Follow up
Fischer *et al*.^25^	PET/CT	350 MBq	60	Static acquisition	Qualitative	1.5	DW-EPI-FS(WB), T2wFIESTA(WB)	7mm	No	Yes (Bvalue0-700mm2/s)	Yes	40	PET/CT
Schmidt *et al*.^26^	PET/CT	294 MBq	60	Static acquisition	Qualitative, semi-quantitative	1.5	STIR(WB), HASTE(abdomen), HASTE(lung), STIR-lung, T2w-FS-TSE(liver), T1wTSE(WB), T1wTSE(spine), STIR(spine), VIBE(liver), T1-FS- GE(pelvis), T1wTSE(brain), T2wTSE(brain)	3–5mm	Yes	No	No	51	Follow up
Cafagna *et al*.^27^	PET/CT	370–550 MBq	60	Static acquisition (3min per bed position)	Qualitative, semi- quantitative	1.5	TSE(WB), DW-EPI-STIR(WB)	5mm	No	Yes (B-value0-500-1000mm2/s)	Yes	51	Follow up
Manenti *et al*.^28^	PET/CT	NR	NR	Static acquisition (4min per bed position)	Qualitative	3.0	T1wTFE(WB), T2wTFE(WB), THRIVE- FFE(WB), DW-EPI-STIR(WB)	4–6mm	Yes	Yes (B-value0-1000mm2/s)	No	35	Histology and/or follow up
Punwani *et al*.^29^	PET/CT	370 MBq	60	Static acquisition	Qualitative, semi-quantitative	1.5	STIR-RARE(WB)	7mm	No	No	No	25–30	PET/CT
van Ufford *et al*.^30^	PET/CT	3 MBq/kg	60	Static acquisition (3min per bed position)	Qualitative	1.5	T1wTSE(WB), T1wSTIR(WB), DW-EPI(head, neck), DW-EPI-FS(chest, abdomen, pelvis)	6mm	No	Yes (Bvalue0-1000mm2/s)	No	55	Follow up
Abdulqadhr *et al*.^31^	PET/CT	5 MBq/kg	60	Static acquisition (3min per bed position)	Qualitative	1.5	T1wTSE(WB), T2wSTIR- FS, DWIBS(WB), T2wTSE, T1wGE(chest, abdomen)	6mm	No	Yes (Bvalue0-1000mm2/s)	No	50	Histology and/or follow up
Gu *et al*.^32^	PET/CT	4.8 MBq/kg	60	Static acquisition (4min per bed position)	Qualitative	3.0	T2wSPAIR-FS, DW-EPI-STIR	5mm	No	Yes (B-value0-1000mm2/sec)	No	48	PET/CT
Lin *et al*.^33^	PET/CT	5 MBq/kg	60	Static acquisition (2min per bed position)	Qualitative, semi-quantitative	1.5	DW-EPI-FS(WB)	5mm	No	Yes (Bvalue50-400-800mm2/s)	Yes	30–45	PET/CT
Wu *et al*.^34^	PET/CT	370 MBq	60	Static acquisition (3min per bed position)	Qualitative, semi-quantitative	3.0	T1wTSE(WB), T2wIR(WB), T1wGEVIBE(neck, abdomen), T1wGE-VIBE(neck, abdomen)after CM, T2wTSE(neck, abdomen), T2wTSE-FS(abdomen), DW-EPI(WB)	1–5mm	Yes	Yes (Bvalue0-800mm2/s)	Yes	27	Follow up
Chen *et al*.^35^	PET and PET/CT	NR	NR	NR	NR	1.5	DW-EPI-STIR, FSE	6–7mm	No	Yes (B-value0-800mm2/s)	Yes	43	Histology
Shortt *et al*.^36^	PET/CT	250–440 MBq	90	Static acquisition	Qualitative, semi-quantitative	1.5	STIR(WB), T1wTSE(WB)	8mm	No	No	No	20	Histology
Daldrup-Link *et al*.^37^	PET	3.7 MBq/kg	60	Static acquisition (4-6min per bed position)	Qualitative	1.5	T1wSE, T2wSTIR-FS	4–6mm	No	No	No	45–60	Histology and/or follow up
Schmidt *et al*.^38^	PET/CT	202–372 MBq	60	Static acquisition (3min per bed position)	Qualitative, semi-quantitative	1.5	STIR(WB), HASTE-STIR(lung), T2wSE(liver), T1wSE(WB), T1w+STIR(spine), 3D-VIBE(liver)after CM, T1wGE-FS(abdomen)after CM, T1w+T2w(skull)	5mm	Yes	No	No	55	Histology and/or follow up
Ribrag *et al*.^39^	PET/CT	539 MBq	46–184	Static acquisition (7–8min per bed position)	Qualitative, semi-quantitative	1.5	STIR(WB), T1wSE(WB)	8mm	No	No	No	20	Histology
Kumar *et al*.^40^	PET/CT	5.2 MBq/kg	45	Static acquisition	Qualitative	1.5	SE-STIR(WB)	NR	No	No	No	40–60	Histology and/or follow up
Takenaka *et al*.^41^	PET/CT	3.3 MBq/kg	60	Static acquisition (2min per bed position)	Qualitative, semi-quantitative	1.5	T1wGE(WB), T1wGE(WB)after CM, Opposed-phase T1 GE(WB), STIR- TSE(WB), DW-EPI-STIR(WB)	8mm	Yes	Yes (Bvalue0-1000mm2/s)	No	75	Follow up
Heusner *et al*.^42^	PET/CT	260 MBq	60	Static acquisition (4–6min per bed position)	Qualitative, semi-quantitative	1.5	T1wGE(chest, abdomen), T2wHASTE(chest, abdomen), T1wVIBE(abdomen)after CM, T1wVIBE(head, chest pelvis) after CM	3–7mm	Yes	No	No	NR	Follow up
Ng *et al*.^43^	PET/CT	370 MBq	50–70	Static acquisition (3min per bed position)	Qualitative	3.0	T2wTSE-FS(head, neck, T1wTSE(head, neck), T1wTSE(spine), STIR(spine)T1wTSE-WB, STIR-WB, T2wHASTE(chest, abdomen), T1wVIBE(abdomen), T1wVIBE(abdomen in artery, portal, equilibrium phase) after CM, T1wVIBE(chest, pelvis) after CM, T1wTSE-FS after CM	3–5mm	Yes	No	No	37	Histology and/or follow up
O‘Neill *et al*.^44^	PET/CT	NR	NR	NR	Qualitative	1.5	NR	NR	NR	No	No	20	NR
Ng *et al*.^45^	PET/CT	370 MBq	50–70	Static acquisition (3min per bed position)	Qualitative	3.0	T2wTSE-FS(head, neck), T1wTSE(head, neck), T1wTSE(spine), STIR(spine), T1wTSE(WB), STIR(WB), T2wHASTE(chest, abdomen), T1wVIBE(abdomen), T1wVIBE(abdomen)after CM, T1wVIBE(chest, pelvis)after CM, T1wTSE-FS after CM	3–5mm	Yes	No	No	37	Histology and/or follow up
Chan *et al*.^46^	PET/CT	370 MBq	50–70	Static acquisition (2min per bed position)	Qualitative, semi-quantitative	3.0	T2wTSE-FS(head, neck), T1wTSE(head, neck), T1wTSE(spine), STIR(spine)T1wTSE(WB), STIR(WB), T2wHASTE(chest, liver), T1wVIBE(abdomen), T1wVIBE(abdomen)after CM, T1wVIBE(chest, pelvis)after CM, T1wTSE-FS after CM	3–5mm	Yes	No	No	50	Histology and/or follow up
Eiber *et al*.^47^	PET/CT	350–500 MBq	90	Static acquisition (2min per bed position)	Qualitative	3.0	Dixon VIBE T1w(WB), T2 STIR(neck), T1 TSE(neck), T1 TSE after CM(neck), T1 TSE FS after CM(neck), VIBE T1w dynamic(liver), VIBE T1w after CM(lungs)	2.6–5mm	Yes	No	No	23	Histology and/or follow up
Plathow *et al*.^48^	PET/CT	360–400 MBq	55–65	Static acquisition (3min per bed position)	Qualitative	1.5	STIR(chest), VIBE-FS	NR	NR	No	No	60	Histology and/or follow up
Ohno *et al*.^49^	PET/CT	3.3 MBq/kg	60	Static acquisition (2min per bed position)	Qualitative	1.5	T1wGE(WB), T1wGE(WB)after CM, Opposed-phase T1wGE(WB), STIR-TSE(WB, DW-EPI-STIR(WB)	NR	Yes	Yes (Bvalue0-1000mm2/s)	No	75	Histology and/or follow up
Yi *et al*.^50^	PET/CT	370 MBq	45	Static acquisition	Qualitative	3.0	T2wTSE-FS(WB), T1wTFE(WB) after CM	4–8mm	Yes	No	No	40	Histology and/or follow up
Chen *et al*.^51^	PET/CT	3.3 MBq/kg	60	Static acquisition	Qualitative	1.5	DW-EPI(WB)	6mm	No	Yes (Bvalue0-1000mm2/s)	No	12	Histology and/or follow up
Pfannenberg *et al*.^52^	PET/CT	370 MBq	55–65	Static acquisition (3min per bed position)	Qualitative, semi-quantitative	1.5	NR	NR	NR	No	No	NR	Histology and/or follow up
Laurent *et al*.^53^	PET/CT	5.5 MBq/kg	60	Static acquisition (3–4 min per bed position	Qualitative	1.5	2D-STIR(WB), 3D-T1w(WB)after CM, DW-EPI(WB)	7–8mm	Yes	Yes (Bvalue0-600mm2/s)	No	60	Histology and/or follow up
Dellestable *et al*.^54^	PET/CT	5.5 MBq/kg	60	Static acquisition	Qualitative, semi-quantitative	1.5	T2wSTIR(WB), T1(WB), DWI(WB), T1w3D-GE(WB)after CM	NR	Yes	Yes (Bvalue NR)	No	60	Histology and/or follow up
Schmidt *et al*.^55^	PET/CT	200 MBq	60	Static acqusition	Qualitative, semi-quantitative	1.5–3.0	STIR(WB), HASTE(abdomen), HASTE(lung), STIR(lung), T2w-SE FS(liver), T1wTSE(WB), T1wTSE(spine), STIR(spine), Dyn. VIBE(liver)after CM, Static VIBE(lung, breast)after CM, T1wGE- FS(pelvis)after CM, T1wSE(brain) after CM, T1wGE(brain), T2wSE(brain)after CM	NR	Yes	No	No	43–52	Histology and/or follow up
Heusner *et al*.^56^	PET/CT	300 MBq	60	Static acquisition(4min per bed position)	Qualitative, semi-quantitative	1.5	DW-EPI(WB), HASTE-FS(spine), DW-EPI(spine), T2wSPAIR(WB), T1wFLASH(WB), T2wHASTE(WB), T1wVIBE(WB)after CM	3–6mm	Yes	Yes (Bvalue50-600-800mm2/s)	Yes	NR	Histology and/or follow up
Squillaci *et al*.^57^	PET/CT	370 MBq	45–60	Static acquisition (4min per bed position)	Qualitative, semi-quantitative	3.0	T1wFFE(WB), T2wTSE(WB), T2wTSE- STIR(WB), THRIVE-SPAIR(WB), T1wFFE(WB)after CM	4–6mm	Yes	No	No	47–55	Histology and/or clinical/imaging follow up
Schmidt *et al*.^58^	PET/CT	197–390 MBq	60	Static acquisition	Qualitative, semi-quantitative	1.5–3.0	STIR(WB), T1wTSE(WB), HASTE(lung), STIR(lung), T2wTSE-FS(liver), STIR(spine), T1wTSE(spine), VIBE(liver)after CM, T1wTSE(brain) after CM, T2wTSE(brain)after CM, T1wGE-FS(abdomen)after CM	1.5–6mm	Yes	No	No	42–51	Follow up
Giraudet *et al*.^59^	PET/CT	5 MBq/kg	60	Static acquisition	Qualitative, semi-quantitative	1.5	T2wFSE(liver), dynamic contrast-enhanced MRI, T1-weighted sequences with fast multiplanar spoiled gradient-recalled echo imaging, STIR(WB), T1wSE(WB)	7mm	No	No	No	NR	Follow up
Takano *et al*.^60^	PET	5 MBq/kg	50	Static acquisition (8min per bed position)	Qualitative	1.5	T1wGE(WB), T2wFSE(WB), DW-EPI-STIR(WB)	4mm	No	Yes (Bvalue 0-1000mm2/s)	No	NR	Histology and/or follow up

NR = not reported; CM = contrast media; DWIBS = diffusion weighted imaging with background body signal suppression; WB = whole-body

**TABLE 3.** Diagnostic performance of PET and WB-MRI in the included studies

Authors	Sensitivity (%)				Specificity (%)				Accuracy(%)			

	PET		MRI		PET		MRI		PET		MRI	

	Pt	Les	Pt	Les	Pt	Les	Pt	Les	Pt	Les	Pt	Les
Antoch *et al*.^17^	NR	NR	NR	NR	NR	NR	NR	NR	NR	NR	NR	NR
Schmidt *et al*.^18^	NR	RS	NR	89	NR	RS	NR	86	NR	RS	NR	88
Komori *et al*.^19^	NR	NR	NR	NR	NR	NR	NR	NR	NR	NR	NR	NR
Li *et al*.^20^	NR	NR	NR	NR	NR	NR	NR	NR	NR	NR	NR	NR
Brauck *et al*.^21^	NR	NR	NR	NR	NR	NR	NR	NR	NR	NR	NR	NR
Yang *et al*.^22^	NR	NR	NR	NR	NR	NR	NR	NR	NR	NR	NR	NR
Stecco *et al*.^23^	NR	RS	NR	87–89	NR	RS	NR	98–99	NR	RS	NR	97–99
Krohmer *et al*.^24^	NR	RS	NR	96	NR	NR	NR	NR	NR	NR	NR	NR
Fischer *et al*.^25^	RS	RS	85(WB-MRI), 88(DWI)	57(WB-MRI), 64(DWI)	RS	NR	81(WB-MRI), 69(DWI)	NR	RS	NR	84(WB-MRI), 83(DWI)	NR
Schmidt *et al*.^26^	94	NR	91	NR	97	NR	88	NR	96	NR	89	NR
Cafagna *et al*.^27^	NR	NR	NR	NR	NR	NR	NR	NR	NR	NR	NR	NR
Manenti *et al*.^28^	NR	RS	NR	96(WB-MRI), 94(DWI)	NR	RS	NR	100(WB-MRI), 100(DWI)	NR	RS	NR	97(WB-MRI), 96(DWI)
Punwani *et al*.^29^	NR	100 (nodal) 96(extranodal)	NR	98(nodal) 91(extranodal)	NR	100(nodal) 100(extranodal)	NR	99 (nodal) 99 (extranodal)	NR	100 (nodal) 100(extranodal)	NR	99 (nodal) 99(extranodal)
van Ufford *et al*.^30^	NR	NR	NR	NR	NR	NR	NR	NR	NR	NR	NR	NR
Abdulqadhr *et al*.^31^	NR	NR	NR	NR	NR	NR	NR	NR	NR	NR	NR	NR
Gu *et al*.^32^	NR	RS	NR	89(WB-MRI), 97(DWI)	NR	NR	NR	NR	NR	NR	NR	NR
Lin *et al*.^33^	NR	NR	NR	NR	NR	NR	NR	NR	NR	NR	NR	NR
Wu *et al*.^34^	NR	NR	NR	NR	NR	NR	NR	NR	NR	NR	NR	NR
Chen *et al*.^35^	NR	NR	NR	NR	NR	NR	NR	NR	NR	NR	NR	NR
Shortt *et al*.^36^	NR	59	NR	68	NR	75	NR	83	NR	65	NR	74
Daldrup-Link *et al*.^37^	86	90	76	82	89	NR	100	NR	87	NR	87	NR
Schmidt *et al*.^38^	NR	98(N-stage), 82(M-stage)	NR	80(N-stage), 96(M-stage)	NR	83(N-stage), 82(M-stage)	NR	75(N-stage), 82(M-stage)	NR	96(TNM)	NR	91(TNM)
Ribrag *et al*.^39^	100(bone lesions), 29 (bone marrow)	96(bone lesions), 95(bone marrow)	100(bone lesions), 100(bone marrow)	83(bone lesions), 90(bone marrow)	NR	NR	NR	NR	NR	NR	NR	NR
Kumar *et al*.^40^	NR	90	NR	97	NR	100	NR	99	NR	98	NR	99
Takenaka *et al*.^41^	96	97	64(WB-MRI), 96(DWI)	73(WB-MRI), 95(DWI)	86	95	90(WB-MRI) 79(DWI)	96(WB-MRI), 94(DWI)	88	95	84(WB-MRI), 83(DWI)	95(WB-MRI), 94(DWI)
Heusner *et al*.^42^	45	NR	64	NR	99	NR	94	NR	94	NR	91	NR
Ng *et al*.^43^	87	87	91	89	90	96	91	97	89	95	91	96
O‘Neill *et al*.^44^	NR	NR	NR	NR	NR	NR	NR	NR	NR	NR	NR	NR
Ng *et al*.^45^	72	71	55	64	94	96	90	96	86	92	76	91
Chan *et al*.^46^	NR	81	NR	62	NR	99	NR	99	NR	99	NR	98
Eiber *et al*.^47^	NR	NR	NR	NR	NR	NR	NR	NR	NR	NR	NR	NR
Plathow *et al*.^48^	92(T-stage), 96(N-stage), 100(M-stage)	NR	100(T-stage), 88(N-stage), 100(M-stage)	NR	100(T-stage), 100(N-stage), 100(M-stage)	NR	100(T-stage), 96(N-stage), 100(M-stage)	NR	NR	NR	NR	NR
Ohno *et al*.^49^	62–70	NR	56–60(WB-MRI), 57–67(DWI)	NR	94	NR	92(WB-MRI), 88(DWI)	NR	88–90	NR	86(WB-MRI), 82-84(DWI)	NR
Yi *et al*.^50^	48	NR	52	NR	96	NR	94	NR	86	NR	86	NR
Chen *et al*.^51^	NR	98	NR	91	NR	98	NR	92	NR	97	NR	91
Pfannenberg *et al*.^52^	NR	90	NR	80	NR	77	NR	76	NR	87	NR	79
Laurent *et al*.^53^	NR	73	NR	83	NR	93	NR	98	NR	NR	NR	NR
Dellestable *et al*.^54^	NR	74	NR	83	NR	89	NR	96	NR	74	NR	81
Schmidt *et al*.^55^	NR	91	NR	90	NR	90	NR	86	NR	91	NR	91
Heusner *et al*.^56^	75–100	94	66–100	91	94–100	99	0–100	72	93–100	98	30–100	76
Squillaci *et al*.^57^	NR	NR	NR	NR	NR	NR	NR	NR	NR	NR	NR	NR
Schmidt *et al*.^58^	NR	86	NR	72	NR	96	NR	93	NR	91	NR	83
Giraudet *et al*.^59^	NR	NR	NR	NR	NR	NR	NR	NR	NR	NR	NR	NR
Takano *et al*.^60^	NR	NR	NR	NR	NR	NR	NR	NR	NR	NR	NR	NR

NR = not reported; Pt = per patient-based analysis; Les = per lesion-based analysis; DWI = diffusion weighted imaging; WB-MRI = whole body magnetic resonance imaging
